# Encroachment on Water Corridors Drives Farmers–Giraffe Conflicts Along River Tana Ecosystem in Eastern Kenya

**DOI:** 10.1002/ece3.74067

**Published:** 2026-08-03

**Authors:** Mohamed H. Ali, George Muriuki, Abdullahi H. Ali, Harun Gitari

**Affiliations:** ^1^ Hirola Conservation Program Garissa Kenya; ^2^ Somali Giraffe Project Garissa Kenya; ^3^ Department of Agricultural Sciences and Technology, School of Agriculture and Environmental Sciences Kenyatta University Nairobi Kenya

**Keywords:** analytic hierarchy process, ecosystem fragmentation, mapping human–giraffe conflicts, *Prosopis Juliflora*, reticulated giraffes, supplementary feeding, wildlife water corridors

## Abstract

Human–wildlife conflict (HWC) is an escalating global issue that is influenced by climate change, human population growth, and agricultural expansion. In arid and semi‐arid lands (ASALs) such as Garissa County in Kenya, where resource scarcity intersects with expanding human land use, the effects of HWC are particularly severe, threatening the survival of species like the reticulated giraffe ( *Giraffa reticulata* ). We investigated how degradation of historical water corridors contributes to increasing conflicts between farmers and the reticulated giraffe ( *Giraffa reticulata* ). Using field surveys, spatial mapping, and supplementary feeding trials, we assessed (1) the current status and drivers of water corridor degradation, (2) spatial hotspots of conflict risk, and (3) the effectiveness of targeted mitigation strategies, specifically supplementary feeding. Our results show that *Prosopis juliflora* invasion (63.0%), farmland expansion, and riverbank erosion are the primary drivers of corridor degradation along the River Tana Ecosystem in Eastern Kenya. Conflict hotspots were closely associated with areas near water sources (≤ 0.011 km) and intensive croplands. Supplementary feeding trials significantly reduced giraffe incursions into farmlands (*t* = −9.18, *p* < 0.0001). These findings highlight the central role of supplementary feeding in managing human–giraffe coexistence and offer a practical, scalable framework for conflict mitigation in water‐stressed, human‐modified landscapes.

## Graphical abstract

Human–wildlife conflict in Garissa County, Kenya, is intensified by the degradation of historical water corridors, driven mainly by *Prosopis juliflora* invasion, farmland expansion, and riverbank erosion. This study identifies conflict hotspots near water sources and croplands, and shows that supplementary feeding effectively reduces giraffe incursions into farms, offering a scalable solution for mitigating conflict in arid, human‐modified landscapes.

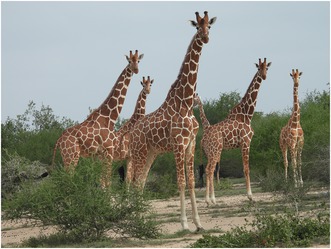

## 1 Introduction

Human–wildlife conflict (HWC) has become an increasingly significant threat to the survival of many endangered species across the globe. According to Distefano (2005), the severity and nature of such conflicts differ from one region to another. Worldwide, the tension between human populations and wildlife is recognized as one of the most complex and pressing challenges faced by conservation efforts (Acharya et al. 2016). Human activities remain the primary danger to wildlife, contributing most significantly to their mortality rates (Nayeri et al. 2022). For example, research conducted in Nepal indicates that rural populations, who rely entirely on farming and raising livestock for their livelihoods, frequently endure financial hardships due to crop destruction or livestock predation by wild animals (Baral et al. 2021).

Conflicts involving humans and mammalian species are currently considered among the most serious threats to wildlife conservation across Africa (Nicole 2019). One major driver of this conflict is crop destruction by wildlife. For example, in Kibale, Uganda, numerous farmers attempt to protect their fields through legal methods such as crop guarding rather than resorting to unlawful activities like hunting (Naughton‐Treves 1998). Comparable incidents of crop damage occur throughout Kenya. In particular, the Masai people of Narok are gradually shifting from their traditional semi‐nomadic herding lifestyle toward agro‐pastoral farming systems (Magembe et al. 2014). The rise in human population has resulted in the transformation of natural wildlife grazing zones (a distinct ecological area characterized by specific environmental or vegetation features that differentiate it from surrounding areas) into agricultural fields, where crops like maize and wheat, commonly targeted and damaged by wildlife, are predominantly cultivated (Mukeka et al. 2019). Other than agriculture, water scarcity also contributes a lot to human–wildlife conflict.

Water is a fundamental resource vital for the survival of both human and wildlife populations. Freshwater ecosystems, rivers, lakes, and wetlands are essential in sustaining biodiversity, agricultural productivity, and local livelihoods (Vörösmarty et al. 2018). However, these systems are increasingly threatened by anthropogenic pressures, unsustainable land use, and climate change (IPBES 2019). These impacts are particularly acute in arid and semi‐arid regions such as Africa, where water scarcity is worsening due to prolonged droughts, erratic rainfall, and the overexploitation of water sources (UNEP 2021). In sub‐Saharan Africa, rivers remain critical for millions who depend on them for drinking water, irrigation, and livestock sustenance (Boretti and Rosa 2019). Unfortunately, deforestation, infrastructure development, and land encroachment have compromised these ecosystems, intensifying human–wildlife conflict (Abrahms 2021).

East Africa is especially vulnerable due to its rapid population growth and expanding agricultural frontiers (Lutta 2023). In Kenya, the Tana River Basin illustrates this crisis. As the country's longest river, the Tana supports millions of people by providing water for domestic use, agriculture, hydropower generation, and wildlife conservation (Njeru 2024). Yet, its ecological integrity is threatened by unsustainable land practices, invasive species, and climate‐induced droughts (Njeru 2024). In Garissa County, the Tana River is a lifeline in a region marked by low and unpredictable rainfall (Ali et al. 2023; Terer et al. 2004). Traditionally home to nomadic pastoralists, this area has witnessed a shift toward sedentary farming due to climate‐related livestock losses, which has led to increased agricultural expansion (Johansson 1991; Nungula et al. 2024; Mohamed 2024). While farming has bolstered food security, it has also fragmented habitats, restricted wildlife corridors (a movement pathway used by both wildlife and humans to access water), and impeded access to water for species such as the reticulated giraffe ( *Giraffa camelopardalis reticulata* ) (Muthiuru et al. 2024).

The giraffe is among the most impacted species in this evolving landscape. As forage becomes scarce, home rangers increase (McQualter et al. 2015), and giraffes increasingly venture into farmlands in search of food, sparking tensions with local communities (Nishad et al. 2025; Njueini 2021). Once seen as gentle and iconic wildlife, giraffes in Garissa are now viewed as agricultural pests, with some farmers resorting to crude and violent deterrents to protect their crops (Ali et al. 2026; Martin 2012). These confrontations are driven by reduced access to water corridors, now obstructed by farms, settlements, and invasive species such as *Prosopis juliflora* (Mutia et al. 2021; Mwalewa et al. 2022; Ali et al. 2023; Cheruto et al. 2024). These environmental changes have far‐reaching consequences for biodiversity, ecosystem stability, and local livelihoods. The persistent degradation of these corridors represents a critical flashpoint in the broader struggle for shared resources between humans and wildlife.

Despite the growing conflict, institutional support has been inadequate. For instance, while compensation is provided for livestock losses caused by lions, giraffes are not included in such schemes, leaving affected farmers feeling marginalized (Bauer et al. 2017). This has led to underreporting of crop damage and increased retaliatory attacks against giraffes (Ali et al. 2023). Supplementary feeding has emerged as a potential mitigation strategy. By providing designated feeding areas, giraffes can be diverted away from farms, minimizing crop destruction. Comparable programs have succeeded with elephants in South Africa and deer in the United States (Ukamaka et al. 2017). However, such interventions must be managed carefully to avoid dependency and preserve natural foraging behaviors (Benka 2023).

Although water access corridors are ecologically significant, there has been limited research on how their degradation fuels localized patterns of human–giraffe conflict. Our study addresses this gap through a multidisciplinary approach combining spatial modeling, field assessments and a supplementary feeding experiment. We were guided by the following research questions:

- How significantly have historical water corridors in Garissa County been degraded, and what are the primary drivers of this degradation?
- Where are the hotspots of human–giraffe conflict, and how do they relate with proximity to water corridors and land‐use change?
- Can targeted mitigation strategies, such as supplementary feeding, effectively reduce giraffe crop‐raiding behavior in high‐conflict areas?

Through these, we aim to inform locally grounded, landscape‐level approaches to reduce conflict and promote sustainable coexistence between wildlife and agricultural communities, especially in arid rangelands.

## 2 Materials and Methods

### 2.1 Study Area

The study was conducted in Garissa County, specifically targeting three villages: Bour Algy, Sankuri, and Kamuthe. Garissa falls within the classification of Arid Land, receiving annual rainfall between 350 and 550 mm, with temperatures soaring up to 40°C during the dry season (Andanje 2002). The county lies predominantly in low‐lying terrain, with elevations ranging from 70 to 400 m above sea level. Its semi‐arid landscape features thorny shrubs, scattered grasslands, and drought‐resistant vegetation, including species of vachellia, *Commiphora*, and *Cenchrus*.

The Tana River, which forms Garissa's western boundary, serves as the only permanent natural water source in the region, supporting both human and wildlife populations. Seasonal rivers emerge during the rainy season and provide temporary water sources, but they also disrupt road access. Invasive species such as *Prosopis juliflora* have significantly altered traditional grazing patterns, intensifying competition for water and pasture among wildlife and livestock.

**FIGURE 1:**
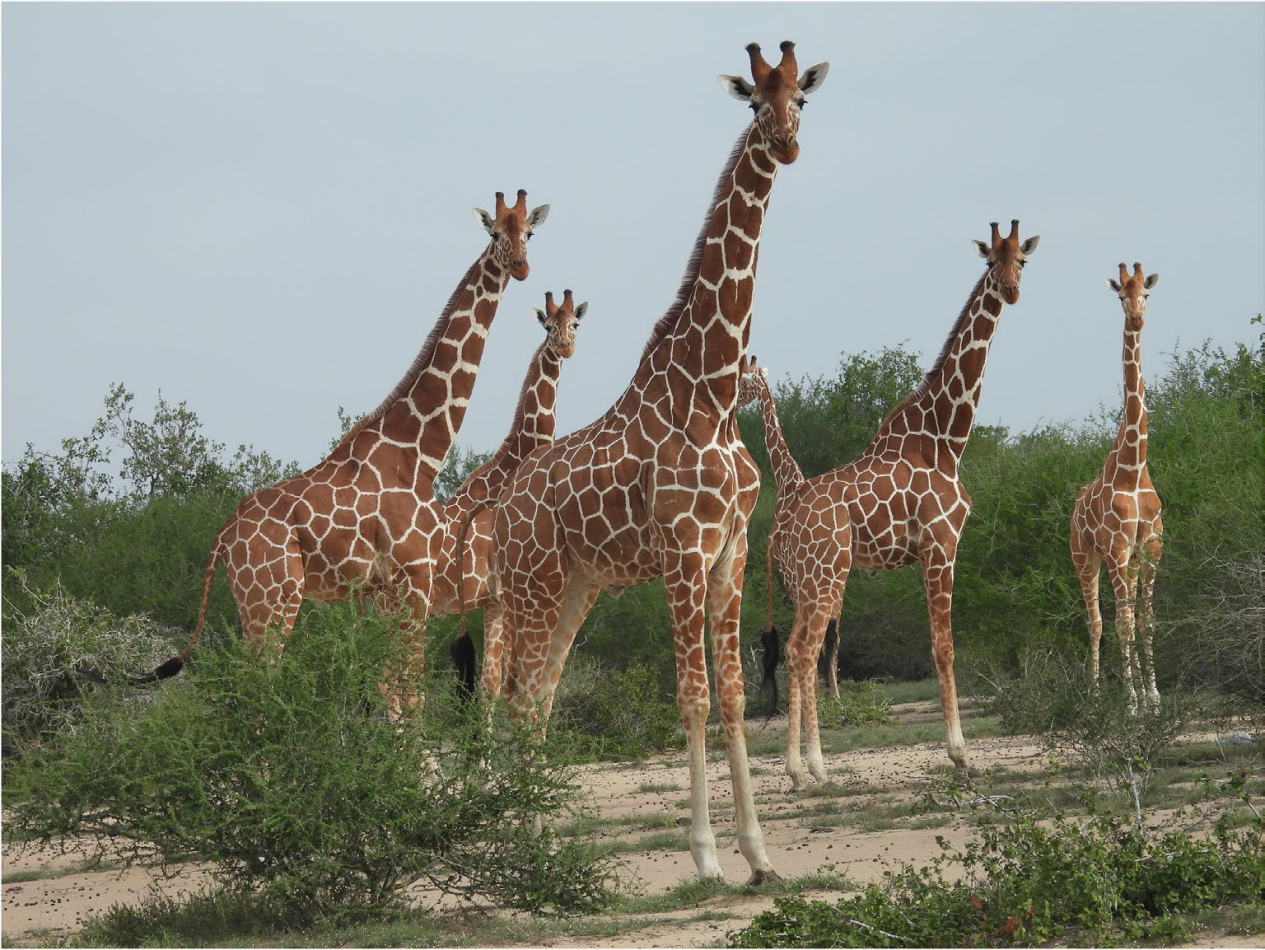
The reticulated giraffe is distinguished by its striking coat pattern of large, polygonal chestnut patches separated by crisp white lines, found across eastern Kenya.

Local Somali pastoralist communities inhabit Garissa and primarily herd camels ( *Camelus dromedarius* ), cattle ( *Bos indicus* ), goats ( *Capra hircus* ), and sheep ( *Ovis aries* ) (Ali et al. 2017). The county also supports a diverse range of herbivore species, including the critically endangered hirola ( *Beatragus hunteri* ), reticulated giraffe ( *Giraffa camelopardalis reticulata* ) (Figure 1), African buffalo ( *Syncerus caffer* ), and lesser kudu ( *Tragelaphus imberbis* ). Large carnivores such as lions ( *Panthera leo* ), leopards ( *Panthera pardus* ), and spotted hyenas ( *Crocuta crocuta* ) help maintain ecological balance in the region.

**FIGURE 2:**
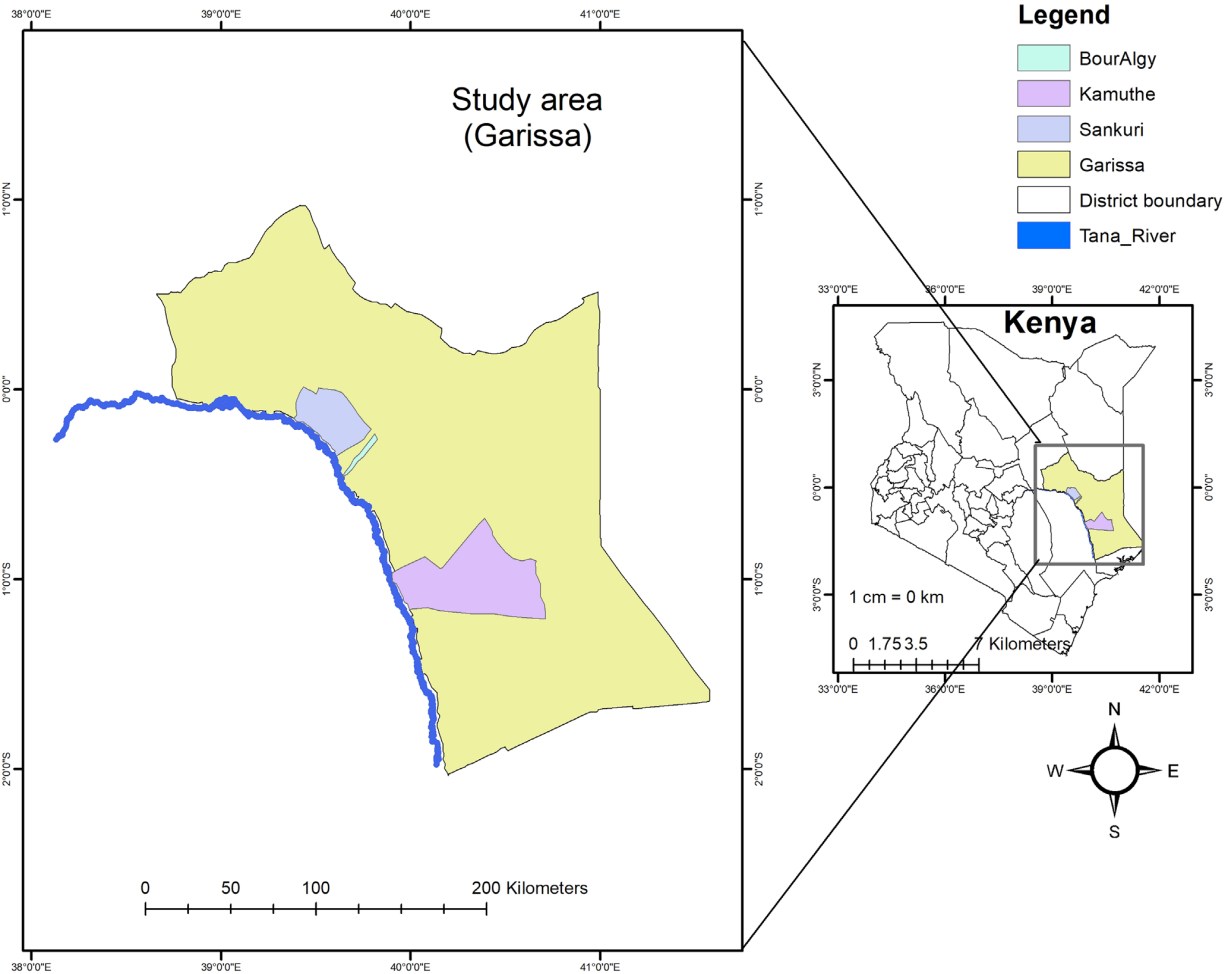
Map of Kenya indicating the study sites in Garissa County and Tana River.

Bird species enrich the region's biodiversity as well, with notable species including Clarke's weaver ( *Ploceus golandi* ) and the saddle‐billed stork ( *Ephippiorhynchus senegalensis* ) (Njoroge et al. 2009). These species, along with the larger fauna and flora, reflect Garissa's ecological significance and its role as a critical habitat in Kenya's arid landscape (Figure 2).

### 2.2 Assessing the Status of Historical Water Corridors

To understand the extent to which historical water corridors in Garissa County have been degraded, and the primary drivers of this degradation, we first conducted surveys along a 50 km stretch of the Tana River, from Sankuri to Kamuthe, where water corridor encroachment and human–giraffe conflicts are prevalent. Using stratified random sampling, we selected 220 households across three zones based on distance from the river (< 1, 1–5, and > 5 km).

We further quantified the extent of *P. juliflora* encroachment using remote sensing, field surveys and Geographic Information Systems (GIS). This was achieved by utilizing Harmonized Sentinel‐2A imagery acquired from the Copernicus Open Access Hub within Google Earth Engine (GEE), a cloud‐based geospatial processing platform, to compute NDVI (Gorelick et al. 2017). We then carried out field surveys to collect GPS coordinates of 20 representative *P. juliflora* locations within the study area, followed by the extraction of their NDVI values and mapping of their distribution in ArcMap version 10.8.

To fully achieve this objective, we generated three diagnostic plots in order to understand (i) which water corridors are most invaded? (ii) are the NDVI classes statistically distinct? and (iii) does water corridor size predict invasion severity? The three include a segmented bar plot of mean NDVI values, boxplot distributions by encroachment class, and a bivariate scatterplot of area versus NDVI. These are meant to provide practical management insights that inform interventions.

### 2.3 Identifying Spatial Hotspots of Conflict Risk

Geographic Information Systems (GIS)‐based Multi‐Criteria Decision Analysis (MCDA) and the Analytical Hierarchy Process (AHP) provide a robust framework for identifying high‐risk conflict zones and have been used to identify high‐risk conflict zones (Malczewski 2006). This objective therefore applies GIS‐based MCDA and AHP to establish spatial hotspots of human–giraffe conflict risk and how they are associated with proximity to water corridors and land‐use change. Analytical Hierarchy Process (AHP) and Geographic Information System (GIS) are in recent times proving to be great approach in assessing and visualizing the relative risk of different areas within a study region. This approach involves integrating various spatial data layers representing risk criteria, conducting pairwise comparisons to establish criteria weights, and developing an aggregated conflict risk map.

We used three variables to establish the probabilities of human–giraffe conflict within our study area:

1. Proximity to Tana River,
2. Proximity to roads,
3. Land Use and Land Cover—Source: Karra et al. (2021).

We reclassified each of the variables using the conflict risk scale in ArcMap version 10.8: very high, high, moderate, low and very low. We then calculated the criteria weightage using pairwise comparison matrix (Figure S1) with AHP. When creating the pairwise comparison matrix, we asked three individuals to compare the three variables/criteria against each other and assign relative importance or preference values to each pair. These values are then represented as elements in the matrix. To ensure the reliability and validity of the pairwise comparison matrix, it is essential to check for consistency. The Consistency Ratio (CR) is a numerical value that measures the degree of consistency in the pairwise comparison matrix. A CR value of < 0.05 (or 5%) is often considered an acceptable level of consistency for a 3 × 3 matrix. If the CR exceeds this threshold, it indicates that the judgments in the pairwise comparison matrix are too inconsistent and may need to be reevaluated. In this objective, the calculated CR is 0.038 (3.8%) indicating that our matrix was consistent and we could proceed to the next step.

The criteria weights derived from the pairwise comparison matrix are as shown in Table 1. Using weighted overlay in ArcMap version 10.8, we mapped and identified the risk of human–giraffe conflict zones within our study area.

**TABLE 1:** Weightage of factors.

Factors	Class
LULC	75.6%
Proximity to river	17.2%
Proximity to roads	7.2%

### 2.4 Testing the Effectiveness of Mitigation Strategies

We conducted a supplementary feeding experiment in Bour Algy (Garissa Giraffe Centre) to assess whether targeted mitigation strategies, such as supplementary feeding, can effectively reduce giraffe crop‐raiding behavior in high‐conflict areas. We randomly selected 27 farms, with 9 farms assigned to each of the three study areas. In Bour Algy (the supplemented group), we provided reticulated giraffes with vachellia pods, sourced from local vendors, at a rate of one 50 kg bag per five adult giraffes. We placed these pods in raised feeding troughs (1.5 m high), which we designed with natural camouflage to blend into the environment. We also supplied water at the feeding stations to further encourage giraffe use. Feeding occurred once daily in the morning (6:00–9:00 a.m.) to align with the giraffes' natural feeding patterns (Pellew 1984; Paulse et al. 2023).

We recorded the number of days giraffes were observed on each farm per month as giraffe presence and the number of days they were not observed as giraffe absence. We collected these data during both the dry and wet seasons to capture potential seasonal variations in giraffe activity. By restricting supplementary feeding to Bour Algy, we established a clear basis for comparison between supplemented and non‐supplemented areas. We conducted all statistical analyses using RStudio (R Core Team 2023). We first checked the data for normality using the Shapiro–Wilk test and assessed homogeneity of variances with Levene's test. Since the data did not violate assumptions of normality and homoscedasticity, we applied the following parametric tests:

- Effect of Supplementary Feeding: We performed an independent samples *t*‐test to compare the mean number of days giraffes were present on supplemented versus non‐supplemented farms.
- Seasonal Variation in Giraffe Presence: We conducted a two‐way analysis of variance (ANOVA) to examine the main effects of season (dry vs. wet) and supplementation (supplemented vs. non‐supplemented) on giraffe presence. We included an interaction term (Season × Supplementation) to test whether the effect of supplementation varied between seasons. We further performed post hoc pairwise comparisons using Tukey's Honestly Significant Difference (HSD) test to determine significant differences between seasonal means.
- Location‐Specific Differences: We performed a one‐way ANOVA to assess differences in giraffe presence across the three locations (Bour Algy, Sankuri, and Kamuthe). Following a significant main effect, we applied Tukey's HSD test to identify specific pairwise differences among the locations.

We used a significance level of *α* = 0.05 for all tests and the ggplot2 package for data visualization in RStudio (Wickham 2016). We reported our results with corresponding test statistics (*F*, *t*, *p*‐values) and confidence intervals where applicable.

## 3 Results

### 3.1 The Current Encroachment Status of Water Access Corridors

A total of 220 people participated in the study with all respondents confirming knowledge of the existence of water access corridors. Respondents reported that most *malkas* or water access points (42.2%) were partially closed, while a smaller portion (13.5%) was fully closed—a difference that was statistically significant (*χ* ^2^ = 24.12, df = 3, *p* < 0.0001). Most respondents also noted that the water points were located on their own properties, whereas a few reported that the points were more than 15 km away, with this distribution showing a significant difference (*χ* ^2^ = 78.96, df = 3, *p* < 0.0001).

We found that 69.9% of respondents confirmed the watering points remained operational, and 45.1% stated these points had been in use for over 25 years. Respondents identified the spread of *Prosopis juliflora* as the leading cause of water point loss (63.0%), followed by farmland conversion, riverbank erosion from the Tana River, and transformation into settlements (Figure S2). These causes differed significantly (*χ* ^2^ = 278.12, df = 6, *p* ≤ 0.0001). Most participants expressed dissatisfaction over the loss of these critical water sources.

We then proceeded to establish the encroachment extent of *P. juliflora* along the water corridors. Foremost, we computed NDVI using Sentinel‐2A imagery within Google Earth Engine (GEE; with a 10 m spatial resolution and 10‐day revisit cycle). The dataset was filtered for February 2022, which improves detection of the invasive species since it is a dry season month when vegetation contrast is maximized. The NDVI values ranged from −0.263 to 0.828, constituting a wide scale varying from barren land or water bodies (negative values) to dense vegetation (higher positive values). The highest NDVI values were concentrated along the Tana River and areas with dense invasions of *Prosopis juliflora* .

The NDVI map (Figure 2a) reveals well defined spatial patterns, with the riverbanks having high vegetation density and the surrounding semi‐arid rangelands exhibiting low NDVI values. The dense *P. juliflora* stands exhibited NDVI values between 0.6 and 0.8, indicative of vigorous vegetation cover. This finding is consistent with previous studies that have used NDVI to map invasive species in semi‐arid ecosystems (e.g., Oumar and Mutanga 2014). We imported The NDVI image into ArcMap version 10.8, where we extracted the NDVI values for the 20 *Prosopis juliflora* locations. These values ranged from 0.368 to 0.766, with a mean of 0.639 indicating moderate to high level of vegetation greenness. The standard deviation of NDVI, 0.117, indicates moderate spatial variability in vegetation density. This shows that there are distinct patches of *P. juliflora* occurring with other vegetation types within the study area. Figure 2b represents the distribution of *Prosopis juliflora* within our study area.

We then extracted the NDVI values of the different water corridors within the study area. The results provide insights into density, spatial distribution, and levels of encroachment across different sections of the corridors. Table 2 represents 19 water corridors with varying characteristics including:

- Mean NDVI across all water corridors is 0.344 (range: 0.190–0.521) indicating moderate vegetation cover.
- Highest mean NDVI is 0.52 (Zone 18). The higher NDVI values suggest dense vegetation, possibly *Prosopis juliflora* encroachment.
- Lowest mean NDVI: 0.19 (Zone 15).
- Standard deviation range is 0.069 to 0.179, indicating moderate to high variability within the water corridors. The higher STD values suggest a mix of open and encroached areas.

**TABLE 2:** Summary statistics of NDVI for the different water corridors.

Corridor ID	Count	Min NDVI	Max NDVI	Mean NDVI	Standard deviation	Total NDVI sum
0	319	0.095	0.481	0.206	0.072	65.774
1	116	0.067	0.706	0.422	0.179	48.997
2	213	0.093	0.748	0.418	0.169	89.123
3	434	0.082	0.590	0.237	0.116	103.244
4	549	0.083	0.711	0.408	0.146	224.412
5	365	0.093	0.716	0.353	0.175	129.183
6	463	0.062	0.620	0.355	0.133	164.642
7	208	0.099	0.637	0.284	0.143	59.270
8	296	0.064	0.653	0.282	0.100	83.505
9	370	0.105	0.711	0.417	0.144	154.412
10	386	0.152	0.763	0.433	0.142	167.415
11	270	0.167	0.677	0.482	0.091	130.174
12	272	0.065	0.654	0.291	0.153	79.222
13	238	0.142	0.665	0.362	0.142	86.276
14	121	0.100	0.559	0.291	0.137	35.278
15	48	0.098	0.314	0.190	0.069	9.141
16	226	0.125	0.597	0.360	0.113	81.547
17	108	0.124	0.384	0.208	0.070	22.540
18	273	0.133	0.767	0.521	0.164	142.290

We then classified the extracted NDVI values into three categories based on previous ecological studies (Kumar and Mutanga 2018; Table 3).

**TABLE 3:** Zonal classification of levels of *P. juliflora* encroachment based on NDVI range.

Classification	NDVI range	Number of zones	Representative zones
Low vegetation cover	≤ 0.3	4 (21%)	Zones 11, 15, 17
Moderate vegetation cover	0.3–0.5	11 (58%)	Zones 0, 3, 6, 7, 8, 12, 13, 14, 16
High vegetation cover	> 0.5	4 (21%)	Zones 1, 2, 4, 5, 9, 10, 18

The High vegetation zones (NDVI > 0.5) have the largest pixel counts (e.g., Zone 10 with 386 pixels) and total NDVI sums (e.g., Zone 4 with SUM = 224.41). Zone 18 showed the maximum single‐pixel NDVI value (0.767) and highest standard deviation (0.165), indicating patches but with dense vegetation. The low vegetation zones had smaller areas (e.g., Zone 15 with only 48 pixels) and lower variability (STD = 0.069). In addition, a positive correlation (*r* = 0.72) was observed between zone area (COUNT) and total NDVI sum (SUM). High vegetation zones accounted for 62% of the total NDVI sum despite representing only 21% of zones.

### 3.2 Analysis of Diagnostic Plots

This analysis classified 19 water corridor segments into three encroachment levels based on NDVI (Table 4). The moderate encroachment level is the dominant one covering 52.6%, indicating widespread but not yet critical infestation. It is followed closely by the low encroachment segments (42.1%) that may still require monitoring to prevent progression. Only one segment (5.3%) shows high encroachment, suggesting an urgent need for targeted intervention.

**TABLE 4:** Corridor counts based on encroachment levels.

Encroachment level	Number of segments	Percentage of total segments
Low (NDVI ≤ 0.3)	8	42.1%
Moderate (0.3 < NDVI ≤ 0.5)	10	52.6%
High (NDVI > 0.5)	1	5.3%

The low‐encroachment segments have NDVI consistently below 0.3, as indicated by the bar plot (Figure S3) indicating sparse vegetation, possibly early‐stage Prosopis or other low‐density species. The Moderate segments, with NDVI 0.3–0.5, have 40% higher vegetation density than low‐encroachment areas, suggesting active Prosopis spread. The single high‐encroachment segment, with NDVI = 0.521, is twice as dense as low‐encroachment zones, confirming severe infestation.

The water corridors exhibiting low encroachment levels have a tight NDVI distribution (Figure S4). This indicates uniform sparse vegetation, while the corridors within the moderate encroachment levels exhibit wider variability (0.3–0.5), implying patchy Prosopis growth. Finally, the one water corridor with high encroachment levels is an outlier (NDVI = 0.52), that is very likely to be a fully invaded zone.

There is a positive relationship between NDVI and area as seen from the scatterplot (Figure S5). This pattern varies based on encroachment levels:

- Low encroachment (Blue points): Generally lower NDVI values (~0.20 to 0.30).
- Moderate encroachment (Orange points): NDVI values fall within a mid‐range (~0.30 to 0.45).
- High encroachment (Red point): Highest NDVI value (~0.50), indicating more vegetation.

### 3.3 Mapping High Risk Human–Giraffe Conflict Zones Using GIS Based Multi‐Criteria Decision Analysis and Analytical Hierarchy Process (AHP)

The integration of AHP‐weighted criteria in GIS produced a high‐resolution conflict risk map, categorizing zones into five classes (Figure S6):

1. Very high‐risk (8% of study area):
  - These areas have immediate proximity to water points (≤ 0.011 km) and intensive croplands and strongly correlate conflict incidents. They also exhibit the highest overlap of giraffe movement corridors and human activity.
2. High‐risk (12%):
  - These areas are adjacent to water points (0.011–0.023 km) and mixed cropland‐rangeland interfaces. They are secondary hotspots for crop raiding and resource competition.
3. Moderate‐risk (22%):
  - These are mostly rangelands and intermediate‐distance buffers from rivers (0.023–0.035 km). Occasional conflicts occur within these areas linked to seasonal resource scarcity.
4. Low‐risk (40%):
  - These are areas beyond 0.035 km from rivers and ≥ 0.020 km from roads. They exhibit minimal human–giraffe interactions and are majorly dominated by natural vegetation.
5. Very low‐risk (18%):
  - These are areas distal to anthropogenic features (roads ≥ 0.030 km) and built‐up areas. They mostly have negligible conflict history and are least prioritized for mitigation.

The Low‐risk class covers the largest area (27,424 Ha, ~45%), confirming that most of the study area is at lower conflict risk (Table 5). The Very High‐risk areas make up a small but significant portion (~5705 Ha).

**TABLE 5:** Area covered by each conflict risk class.

Conflict risk class	Area in hectares (Ha)	Percentage area (%)
Very high	5704.961	9.460
High	1722.252	2.856
Moderate	6912.930	11.463
Low	27,424.479	45.477
Very low	18,538.134	30.741

We finally categorized the identified five conflict risk areas into three priority zones for the purpose of management interventions (Table 6; Venkatesh et al. 2020).

**TABLE 6:** The priority zones and their recommended interventions.

Priority zone	Conflict risk areas	Recommendations
Priority Zone 1 (Urgent)	A combination of Very High and High conflict risk areas	Restore highly *P. juliflora* invaded water corridors to redirect giraffes away from farmlands and high‐conflict areas (Especially corridors 2, 4, 10, and 18 that require immediate intervention given their high NDVI values and large areas). Advocate and promote compensation schemes for farmers who experience giraffe‐related crop damage. Advocate for zoning regulations to prevent uncontrolled agricultural expansion into giraffe habitats including historical water corridors along the Tana River. Installing boreholes and water pans ≥ 0.5 km from croplands to reduce giraffe reliance on conflict‐prone water sources. Promoting Non‐Lethal Deterrents such as giraffe‐proof fencing and motion‐activated sprinklers. Tagging and long‐term monitoring of giraffes to predict movement patterns.
Priority Zone 2	Moderate conflict risk areas	Promoting of alternative livelihoods such as eco‐tourism and sustainable farming practices including the cultivation of alternative crops that are less appealing to giraffes and offer higher economic value. Rehabilitating degraded rangelands to support natural giraffe browsing habitats. Incorporating giraffe conservation into local land‐use policies. Promoting rotational grazing to minimize habitat degradation and reduce competition during dry seasons.
Priority Zone 3	A combination of Low and Very Low conflict risk areas	Expanding protected areas including community conservancies to connect giraffe populations across fragmented landscapes. Enforcing anti‐poaching measures to prevent illegal activities. Engaging local communities in conservation programs and wildlife education.

### 3.4 Testing the Effectiveness of Giraffe Supplementary Feeding to Mitigate the Conflict

**FIGURE 3:**
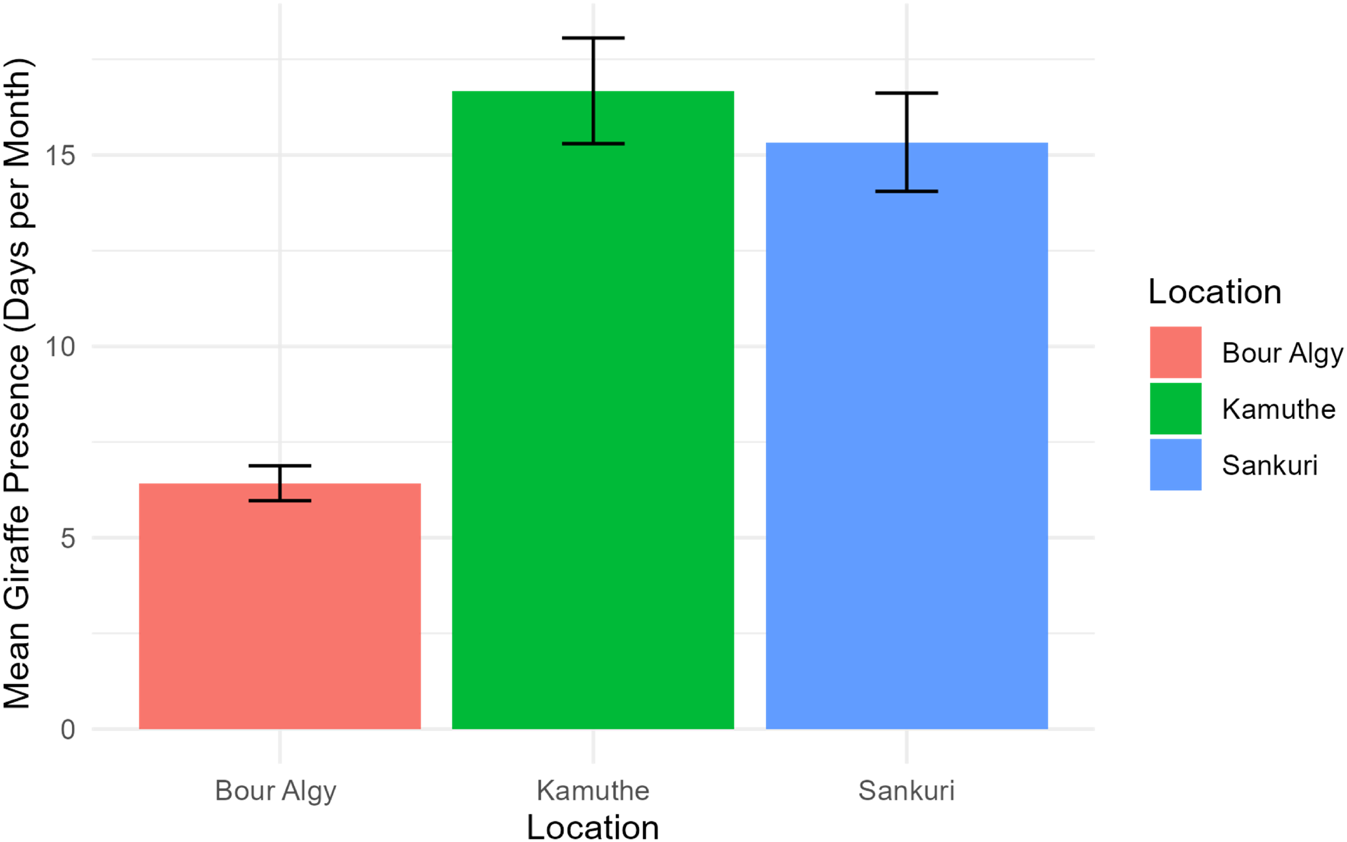
Location based differences in giraffe presence.

The independent samples *t*‐test showed a significant reduction in giraffe presence between Bour Algy as compared to Sankuri and Kamuthe locations (i.e., supplemented farms compared to non‐supplemented farms) (*t* = −9.18, *p* < 0.0001). The supplemented farms in Bour Algy location had a mean presence of 6.42 days/month, whereas non‐supplemented farms in Sankuri and Kamuthe locations had a mean presence of 16.01 days/month. Farms in Kamuthe have the highest giraffe presence, followed closely by Sankuri, while Bour Algy has the lowest (Figure 3). The error bars in the bar chart suggest some fluctuations in the number of giraffes observed at each location. These results imply that supplementary feeding effectively reduces giraffe presence on farms.

**FIGURE 4:**
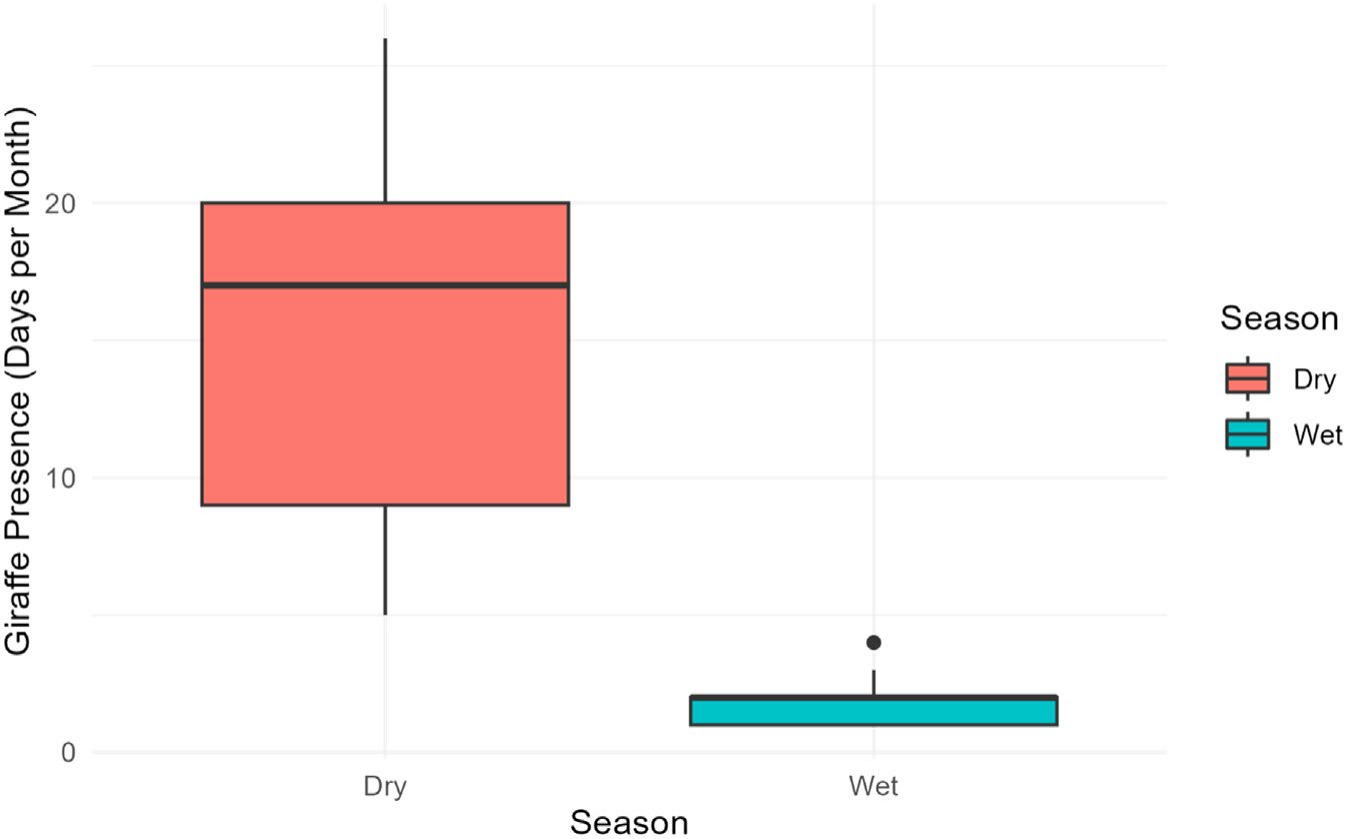
Seasonal variations in giraffe presence.

**FIGURE 5:**
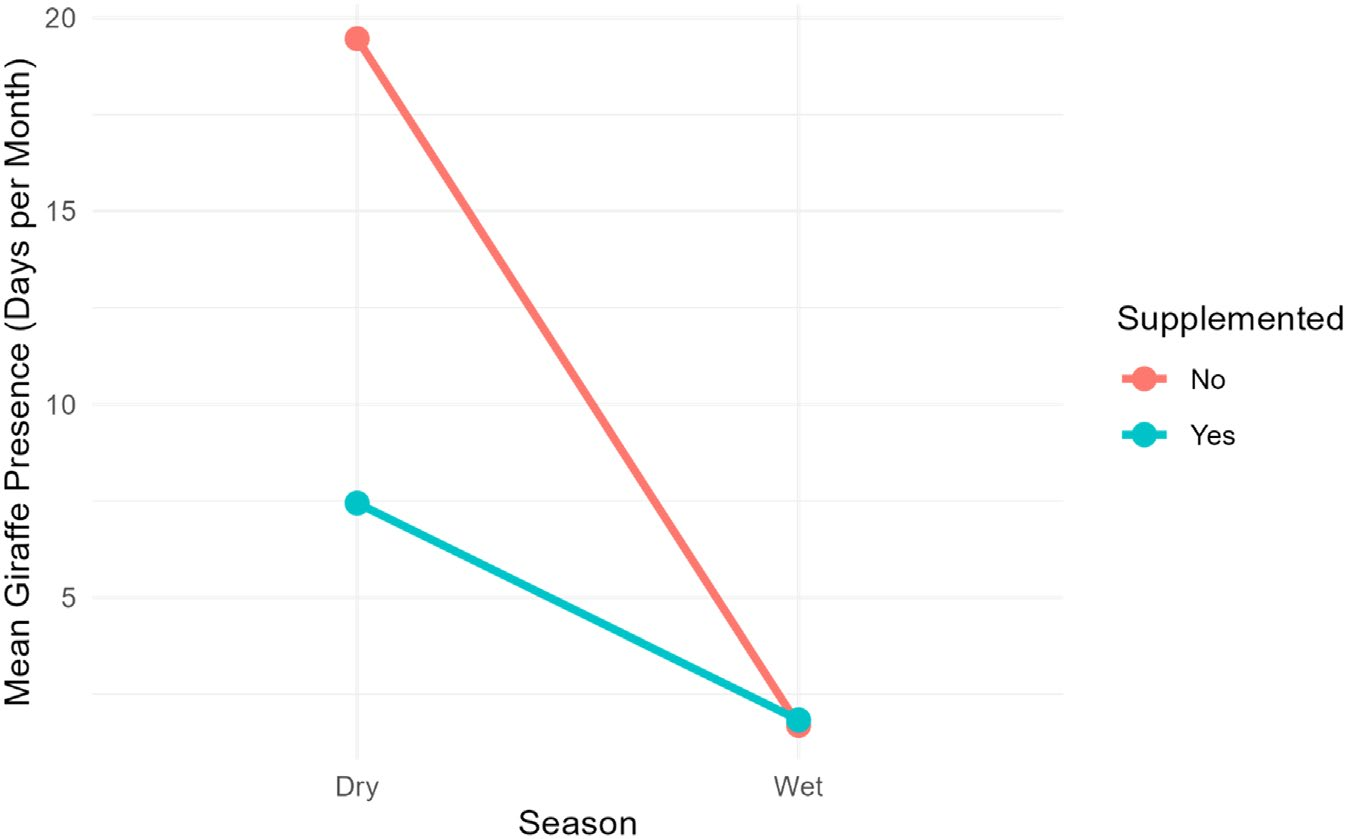
Interaction effects of season and supplementation.

Results from the two‐way ANOVA indicate a highly significant main effect of season (*F* = 405.09, *p* < 0.0001), with giraffe presence in all the locations being substantially lower in the wet season (*M*_wet_ = 1.69 days/month) as compared to the dry season (*M*_dry_ = 19.34 days/month). Additionally, a significant interaction effect between season and supplementation (*F* = 74.10, *p* < 0.0001) implies that the impact of supplementation varies depending on seasonal conditions. This ANOVA result is strongly reinforced by the boxplot (Figure 4) showing seasonal variations (dry vs. wet season). Giraffe presence is significantly higher in the dry season, with a wider spread (more variability), while the wet season has much lower giraffe presence and a more compact distribution. The outliers in the wet season suggest occasional sightings, but giraffes predominantly favor the dry season. Similarly, Figure 5 suggests that giraffe presence in all farms declines sharply in the wet season, regardless of whether supplementary feeding is available. During the dry season, giraffe presence is much higher when there is no supplementary feeding, reinforcing the previous boxplot findings.

**FIGURE 6:**
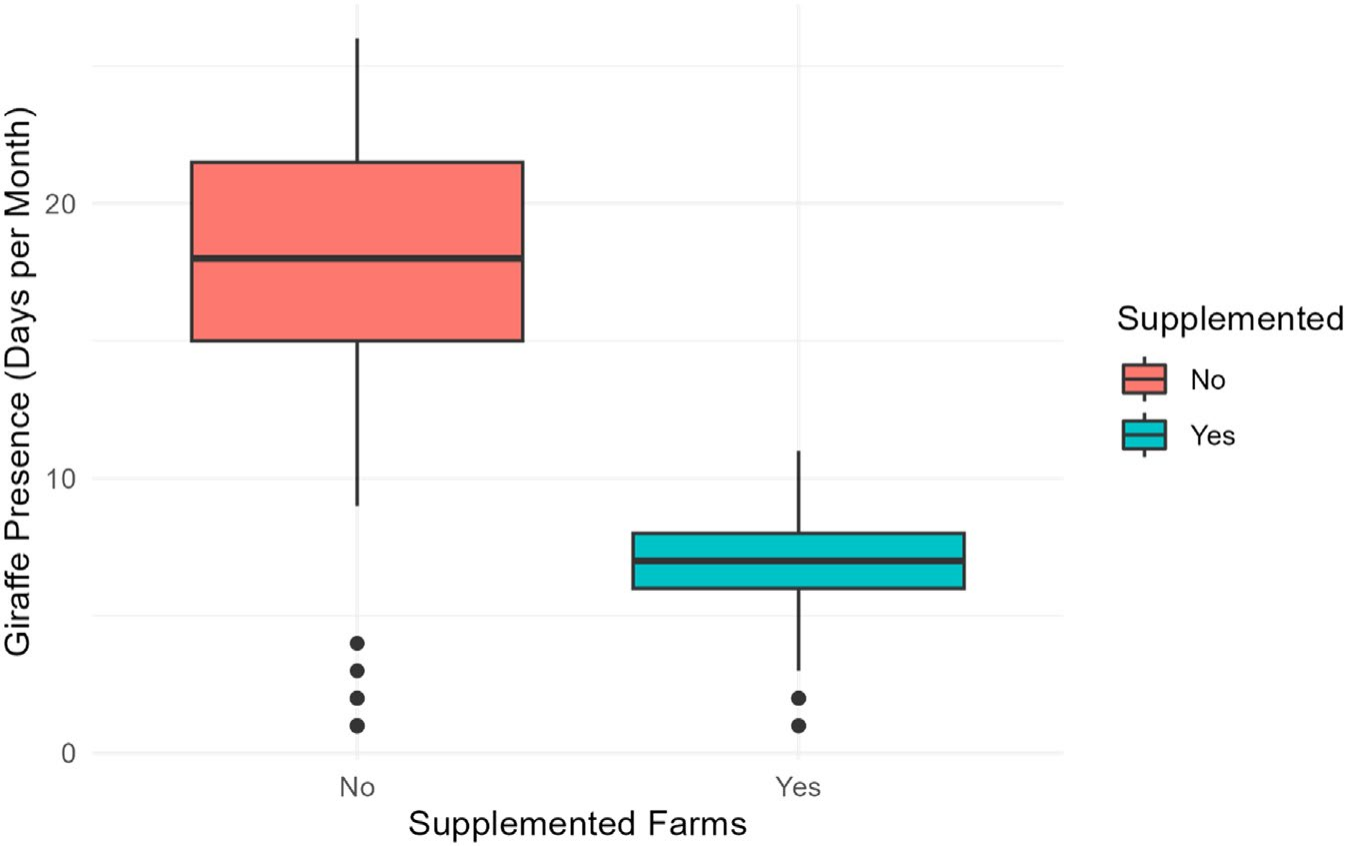
Giraffe presence in supplemented vs. non‐supplemented locations.

The one‐way ANOVA identifies significant differences in giraffe presence across the study locations (*F* = 24.43, *p* < 0.0001). Tukey's post hoc tests indicate that giraffe presence was significantly lower in Bour Algy (where supplementary feeding was provided) compared to Kamuthe (*p* < 0.0001) and Sankuri (< 0.0001). However, no significant difference was found between Kamuthe and Sankuri (*p* = 0.675). Figure 6 reiterates this, indicating higher giraffe presence in areas without supplementary feeding and lower presence where supplementary feeding occurs.

The major findings include:

1. Supplementary feeding significantly reduces giraffe presence on farms.
2. Giraffe presence in farms is significantly lower during the wet season than the dry season.
3. There is a strong interaction between season and provision of feed supplements.

## 4 Discussion

Our study offers new insights on how the degradation of water access corridors, primarily through encroachment of *Prosopis juliflora* and agricultural farms, contributes to human–giraffe conflict in the River Tana ecosystem. A central finding of this study is that *Prosopis juliflora* invasion represents the dominant driver of water corridor degradation in the River Tana ecosystem, blocking access to 63% of water points as reported by community respondents, and thus constituting the primary proximate cause of escalating human–giraffe conflict in the study area. This study demonstrates that the loss of vital water access corridors greatly influences giraffe movements into agricultural farms, increasing both the frequency and severity of crop‐raiding incidents as they search for water and forage, especially during the dry season. These findings highlight the crucial role played by landscape connectivity and access to natural resources in shaping the behavior of herbivores in ASALs.

The degradation of historical water corridors in Garissa emerged as a primary factor influencing giraffe incursions into human‐dominated areas. A majority (63%) of community respondents identified *Prosopis juliflora* , an invasive, fast‐spreading shrub, as the leading cause of corridor degradation. *Prosopis* has been widely reported to displace native flora, reduce palatable forage availability, and form dense thickets that obstruct wildlife movement across many East African rangelands (Shiferaw et al. 2019; Muturi et al. 2020). In Garissa, this transformation is compounded by cropland expansion, often driven by the demand for irrigated agriculture along fertile riparian zones, further fragmenting ecological pathways.

From a management perspective, restoration of degraded water corridors should therefore be prioritized as a central conservation intervention. Restoration efforts should focus on the targeted removal and control of *P. juliflora* , rehabilitation of native browse vegetation, and protection of riparian buffer zones from unregulated agricultural encroachment. Corridors exhibiting high NDVI values and severe invasion levels, particularly Corridors 2, 4, 10, and 18, require urgent intervention. Mechanical clearing combined with reseeding of native drought‐tolerant browse species may help restore corridor functionality while reducing pressure on farmlands. However, because *P. juliflora* reinvasion rates are often high, long‐term community‐based management and continuous monitoring will be essential for restoration success.

This loss of access to traditional dry‐season resources forces giraffes to seek alternative forage, primarily in agricultural lands. Our spatial analysis confirms that conflict hotspots were concentrated near water sources (≤ 0.011 km) and high‐density cropland zones, suggesting a strong spatial link between corridor obstruction and conflict severity. These findings align with studies on large herbivores, including elephants (Graham et al. 2010), which demonstrate that landscape fragmentation and blocked access to key water and forage resources consistently increase the frequency of human–wildlife interactions.

The increasing presence of giraffes in farmlands indicates behavioral shifts driven by habitat modification. Giraffes are known to adjust their movement patterns based on forage availability, water access, and disturbance (Deacon and Smit 2017; Muneza et al. 2016). In Garissa, the decline in native forage within corridors likely contributes to crop‐raiding behavior. Our supplementary feeding experiment that we conducted during the dry season in Bour Algy provides strong proof of this connection. There were fewer crop‐raiding incidents in locations where giraffes received supplementary feeding as compared to where they foraged naturally (*t* = −9.18, *p* < 0.0001). These findings indicate that when dry‐season resource deficits are mitigated, giraffes are less likely to forage on croplands. The study is comparable to evidence from other ungulates that suggested supplementary feeding can be effective when strategically applied. For example, a study on mule deer found that fawn survival was 53% in supplemented groups, while no fawns survived in non‐fed groups, indicating that supplementary feeding can improve survival during harsh conditions. However, the study also noted that long‐term sustainability depends on management costs, logistics, and potential ecological risks (Pal et al. 2026).

This behavior not only reflects the reticulated giraffes' resilience, but also their vulnerability in human‐modified landscapes. Giraffes will continue facing exposures to risks such as retaliatory killings, poaching, and disease transmission from livestock without adequate corridors and buffer zones (Ogutu et al. 2016). Additionally, landscape changes that disrupt movement may have long‐term impacts on gene flow and population viability, particularly for isolated populations like those in Garissa.

Incorporating local knowledge into our study highlights the strong awareness among residents of environmental changes and their impacts. Community members attributed the loss of water corridors to *Prosopis* invasion, expanding farmlands, and natural river erosion; an interplay of ecological and socio‐economic drivers. Similarly, studies from other ASALs areas (King et al. 2017; Kiffner et al. 2020) established that locals acknowledged both their ecological value and role in crop damage.

This dual perception highlights an opportunity for co‐management strategies that align community needs with conservation goals. Participatory restoration of corridors, supported by local institutions, could help reduce conflict while also enhancing landscape functionality. These approaches are especially important in areas like Garissa, where there is a lack of adequate wildlife protected areas, and local communities play a central role in land stewardship.

This study highlights the potential of co‐management strategies including approaches that integrate community governance with conservation objectives as a practical pathway for addressing human–giraffe conflict in areas like Garissa where formal protected areas are absent and customary land governance structures remain strong. The evidence from comparable semi‐arid African settings consistently shows that co‐management succeeds when it creates tangible and equitable benefits for local communities while simultaneously achieving conservation goals, rather than treating these as competing objectives.

One example in the East African context is the Laikipia Plateau in north‐central Kenya, a landscape structurally comparable to Garissa in that it supports large wildlife populations almost entirely outside national parks or government game reserves. Kinnaird and O'Brien (2012) used camera trap data across eight properties in Laikipia to demonstrate that species richness, occupancy, and relative abundance of large mammals were significantly higher on conservancies with intermediate livestock stocking than on fenced commercial ranches or unfenced group ranches with high stocking densities. Critically, they attributed this not solely to reduced livestock pressure, but to the presence of institutional arrangements that gave landowners rights and incentives to tolerate wildlife. This finding is directly relevant to Garissa, where farmers currently receive no compensation for giraffe crop damage and therefore bear the full cost of coexistence. Kinnaird and O'Brien (2012) specifically recommended that national conservation strategies adopt landscape‐level land‐use planning that provides landowners with incentives to tolerate wildlife, rather than relying on exclusionary protected area models. Applied to the Garissa context, this suggests that formalizing community‐led conservation agreements around the Tana River corridor linked to compensation schemes and alternative livelihood support could substantially reduce retaliatory pressures on giraffes.

The Northern Rangelands Trust (NRT) model in northern Kenya offers perhaps the most geographically and ecologically relevant example of co‐management at scale. Established in 2004, NRT now supports over 40 community conservancies spanning more than 10% of Kenya's landmass across arid and semi‐arid counties, including Garissa and Tana River. Community conservancies under NRT are legally registered entities governed by representative boards and locally staffed management teams, with programs spanning wildlife protection, rangeland management, peace and security, and local economic development. Research conducted at Naibunga Community Conservancy in northern Kenya found that large proportions of surveyed households (65%–90%) perceived conservancy‐related improvements in their overall socioeconomic status, security, household income, livestock numbers, and accessibility to grazing resources, suggesting that the model can generate genuine livelihood benefits alongside conservation outcomes. This also demonstrates that community conservancies are particularly effective in areas with strong ethnic governance systems and customary land tenure, conditions that apply directly to the Somali pastoralist communities of Garissa (Boone et al. 2024).

Comparative evidence from Tanzania provides further support, though also important cautionary lessons. Baldus and Cauldwell (2004) documented how Wildlife Management Areas (WMAs) in Tanzania were designed to devolve wildlife revenue and management authority to local communities, and how tourist hunting played a role in funding community development around these areas. However, subsequent analyses revealed that centralized control of hunting revenues created disincentives for genuine devolution of management power to local institutions, resulting in limited community buy‐in and weak conservation outcomes in some WMAs. This experience underscores a critical lesson: co‐management frameworks succeed when communities exercise substantive rather than nominal decision‐making authority, and when the financial benefits of wildlife‐based land use are actually distributed to affected households rather than retained by central government agencies.

Together, these case studies point to four conditions that favor successful co‐management in ASAL contexts comparable to Garissa. First, communities must hold clearly defined rights over land and wildlife resources, providing the institutional foundation for long‐term stewardship. Second, tangible economic benefits through ecotourism revenue sharing, carbon payments, or compensation schemes must accrue directly to households bearing the costs of human–wildlife coexistence. Third, governance structures must be locally anchored, drawing on existing customary institutions such as the clan‐based governance systems of Garissa's Somali communities, rather than imposing externally designed management bodies with limited legitimacy. Fourth, co‐management must be sustained over the long term. The NRT experience indicates that conservancies require a minimum investment horizon of 10 years before measurable wildlife and livelihood outcomes stabilize. The Bour Algy Giraffe Sanctuary, already an emerging institution in this landscape with community consent and NGO support from the Hirola Conservation Program and Somali Giraffe Project, could serve as a nucleus around which a more formally structured co‐management arrangement is built, connecting giraffe conservation to the broader concerns of water access, rangeland health, and food security that motivate the communities living alongside the Tana River corridor.

## 5 Limitations

A key limitation of this study was the inability to rotate supplementary feeding treatments across all three sites. Although baseline comparisons of human–giraffe conflict were conducted prior to the study, logistical constraints prevented the implementation of a fully rotated experimental design. In particular, securing suitable and freely available land for supplementary feeding across all study sites proved challenging.

## Author Contributions

**Mohamed H. Ali:** conceptualization (lead), writing – original draft (lead). **George Muriuki:** conceptualization (supporting), writing – review and editing (supporting). **Abdullahi H. Ali:** conceptualization (supporting), supervision (supporting). **Harun Gitari:** conceptualization (supporting), supervision (supporting).

## Funding

This research was funded by The Explorers Club (Fjallraven Field Grant, 2024_FF_1142777915).

## Ethics Statement

Before collecting data, the authors obtained approval from the National Commission for Science, Technology and Innovation (NACOSTI; Ref. No. 412950) and permission from the Kenya Wildlife Service, relevant local authorities and community leaders to conduct the study.

## Consent

The authors explained the study's purpose and procedures to all participants and informed them that participation was voluntary, that they could withdraw at any time without penalty and that their responses would be kept confidential. The authors obtained informed consent from all participants before data collection and the area chief signed as the community representative to document community consent.

## Conflicts of Interest

The authors declare no conflicts of interest.

## Supporting information


**Figure S1:** Pairwise Comparison Matrix between the conflict risk criteria to determine their relative importance.
**Figure S2:** Respondents' perceptions of the main causes of water point encroachment, with the majority identifying *Prosopis juliflora* (locally known as *Mathenge*) and conversion of land to agriculture as the primary drivers.
**Figure S3:** Bar plot showing NDVI values per segment with color‐coded encroachment levels.
**Figure S4:** Boxplot displaying NDVI distribution across encroachment levels.
**Figure S5:** Bivariate scatterplot of area versus NDVI.
**Figure S6:** Human–Giraffe conflict risk map of our study area.

## Data Availability

The data is available and can be accessed in the manuscript.
