# Supplementary material for: Encroachment on Water Corridors Drives Farmers–Giraffe Conflicts Along River Tana Ecosystem in Eastern Kenya
Source: Ecol Evol. 2026 Aug 3;16(8):e74067. doi: 10.1002/ece3.74067 (PMC13433143; doi:10.1002/ece3.74067)
Supplement: Supplementary file 1 [file ECE3-16-e74067-s001.docx]

**Supplementary Section**

Figure S1: Pairwise Comparison Matrix between the conflict risk criteria to determine their relative importance.

% reponse

| Causes of Malkas (water points) to Non-operational | Percent |
| --- | --- |
| It was invaded by Mathenge | 63.0252 |
| Completly deformed by river making it a cliff | 2.52101 |
| Currently not aware | 0.840336 |
| Converted to oxblow lake | 0.840336 |
| It was converted into farms | 27.7311 |
| It was converted into settlement | 4.20168 |
| It was shut down by the government | 0.840336 |

Figure S2: Respondents’ perceptions of the main causes of water point encroachment, with the majority identifying *Prosopis juliflora* (locally known as *Mathenge*) and conversion of land to agriculture as the primary drivers.

Figure S3: Bar plot Showing NDVI values per segment with color-coded encroachment levels

Figure S4: Boxplot Displaying NDVI distribution across encroachment levels

Figure S5: Bivariate scatterplot of area versus NDVI

Figure S6: Human-Giraffe conflict risk map of our study area.
